# Rare Neural Correlations Implement Robotic Conditioning with Delayed Rewards and Disturbances

**DOI:** 10.3389/fnbot.2013.00006

**Published:** 2013-04-02

**Authors:** Andrea Soltoggio, Andre Lemme, Felix Reinhart, Jochen J. Steil

**Affiliations:** ^1^Faculty of Technology, Research Institute for Cognition and Robotics (CoR-Lab), Bielefeld UniversityBielefeld, Germany

**Keywords:** classical conditioning, instrumental conditioning, distal reward, robotics, neuromodulation

## Abstract

Neural conditioning associates cues and actions with following rewards. The environments in which robots operate, however, are pervaded by a variety of disturbing stimuli and uncertain timing. In particular, variable reward delays make it difficult to reconstruct which previous actions are responsible for following rewards. Such an uncertainty is handled by biological neural networks, but represents a challenge for computational models, suggesting the lack of a satisfactory theory for robotic neural conditioning. The present study demonstrates the use of rare neural correlations in making correct associations between rewards and previous cues or actions. Rare correlations are functional in selecting sparse synapses to be eligible for later weight updates if a reward occurs. The repetition of this process singles out the associating and reward-triggering pathways, and thereby copes with distal rewards. The neural network displays macro-level classical and operant conditioning, which is demonstrated in an interactive real-life human-robot interaction. The proposed mechanism models realistic conditioning in humans and animals and implements similar behaviors in neuro-robotic platforms.

## 1 Introduction

In reward learning, the results of actions, manifested as rewards or punishments, occur often seconds after the actions that caused them. For this reason, it is not always easy to determine which previous stimuli and actions are causally associated with following rewards. This problem was named *distal reward problem* (Hull, 1943), or credit assignment problem (Sutton and Barto, 1998). This problem and the ability of animals to solve it emerged originally in behavioral psychology (Thorndike, 1911; Pavlov, 1927; Skinner, 1953). More generally, the distal reward problem can be seen as a particular instance of the broader ontological problem of discovering apparent cause-effect relationships in the external world. The ability of determining such relationships is distinctive of human and animal intelligence.

Such abilities were observed for example by Pavlov (1927), who induced a dog to believe that the ringing of a bell predicted the arrival of food. After conditioning, the ringing of the bell alone triggered salivation. Thorndike (1911) was also the first to describe how animals learn from experience which course of actions leads to best outcomes. Even organisms with relatively simple neural systems, like the marine mollusk Aplysia, are capable of associating neutral stimuli with following noxious stimuli in classical (Kandel and Tauc, 1965; Carew et al., 1981) and operant conditioning (Brembs et al., 2002). The capability of discovering relationships among stimuli, actions, and rewards in the world is therefore not a prerogative of human cognition, but it is also largely exploited in animal intelligence. Such a notion implies that relatively basic neural dynamics, as those of the Aplysia, can associate stimuli, actions, and reward across time and lead to what can be seen as a primordial version of temporal inductive inference (Osherson et al., 1990).

An important topic in neural computation is the understanding of how small neural networks discover relationships among events, even in the presence of interfering stimuli, or considerable time delays between cues, actions, and outcomes. One hypothesis that has gathered consensus in the last decade is that of *synaptic tagging* (Frey and Morris, 1997; Redondo and Morris, 2011) or *eligibility traces* (Wang et al., 2000; Sarkisov and Wang, 2008). The idea is that particular neural events, deriving for example from performing an action or perceiving a cue, leave slowly decaying traces in the network. The traces expire for unrelated and disturbing stimuli, but get promoted to long term synaptic changes when a reward follows. The utility of synaptic tags in the solution of the distal reward problem was shown in simulation in Päpper et al. (2011).

Conditioning occurs with the delivery of rewards or punishments in the form of pleasant or noxious stimuli. Reward signals were found to be mediated both in vertebrate and invertebrate organisms by neuromodulation (Carew et al., 1981; Hammer, 1993; Schultz et al., 1993; Menzel and Müller, 1996). The increasing evidence of the important role of neuromodulation in reward-driven learning led to the formulation of models of modulated plasticity with rate-based neurons (e.g., Montague et al., 1996; Alexander and Sporns, 2002; Sporns and Alexander, 2002; Ziemke and Thieme, 2002; Soltoggio et al., 2008; Soltoggio and Stanley, 2012), and with spiking neurons and modulated spike-timing-dependent-plasticity (STDP) (Soula et al., 2005; Farries and Fairhall, 2007; Florian, 2007; Legenstein et al., 2008; Potjans et al., 2009, 2011; Vasilaki et al., 2009). This evidence suggests that neuromodulation is both a biological (Schultz et al., 1993, 1997; Hasselmo, 1995) and a computational (Montague et al., 1996; Porr and Wörgötter, 2007) effective medium to convey reward information to a neural substrate. Neuromodulation, however, involves a variety of modulatory chemicals, which are observed to regulate a spectrum of neural functions, from arousal to attention, exploration, exploitation, memory consolidation, and other (Hasselmo, 1995; Marder and Thirumalai, 2002; Aston-Jones and Cohen, 2005). The implementation of such functions is investigated in a number of computational models (Fellous and Linster, 1998; Doya, 1999, 2002) and neural robotic controllers (Krichmar, 2008; Cox and Krichmar, 2009), in particular with focus on the role of neuromodulation in attention (Avery et al., 2012).

Relatively few studies focus on the particular neural mechanisms that bridge the temporal gap between sequences of cues, actions, and rewards (Izhikevich, 2007; Päpper et al., 2011; Soltoggio and Steil, 2013). In Izhikevich (2007), the precise spike-timing of neurons was indicated as the essential feature to perform classical and operant conditioning with modulated STDP. This position was challenged in a recent study (Soltoggio and Steil, 2013) in which the *rarity* of both correlating neural activity and eligibility traces was identified as the main feature that allowed for the solution of the distal reward problem also in rate-based models. The rarity of correlations was shown in simulation to be responsible for selecting rare neural events. Such events are then propagated further in time and enable weight updates if rewards occur.

The identification of the neural principles that solve the distal reward problem is fundamental in understanding how biological networks find relationships among stimuli and improve behavioral responses over time. Robots provide a realistic means for testing computational models that deal with similar timing and complexity of sensory information as those of living organisms. Cognitive developmental robotics (Asada et al., 2001), for example, is an area in which human feedback is used during learning. In such contexts, the asynchrony of flows of inputs and outputs implies that a learning neural network must cope with imprecise timing and unreliability of signals and actions. When people provide cues and feedback in a human-robot interaction, different operators, errors, and disturbances create a complex input-output pattern from which to extract correct relationships among stimuli and actions.

The principle of rare correlations, first introduced in Soltoggio and Steil (2013), is tested in the current study precisely in robotic scenarios in which learning is guided by human feedback. Classical and operant conditioning are tested in a setting in which a neural network serves as controller. Inputs from the robot cameras (the eyes) and tactile sensors (on the hands) are processed by a neural network, which in turn controls robotic actions like displaying a smiling expression, recognizing the tutor and learning to identify the correct color of objects. The learning is guided by the rewards given by the human participants, specifically the tutor, who interacts with the robot in a natural and spontaneous way, thereby affecting the robot perception with uncertain timing, delayed reward and disturbances. The successful achievement of conditioning and of behavior reversal proves the validity of the method to simulate realistic conditioning with the proposed neural model.

This paper is organized as follows. The principle of rare correlations and the plasticity mechanism are explained in Section 2. The robotic experimental settings, the conditioning problems and the details of the learning networks are illustrated in Section 3. The results, including both robotic runs and simulations, are presented in Section 4 and discussed in more detail in Section 5. The paper ends with concluding remarks in Section 6. An appendix provides further implementation details.

## 2 Using Rare Correlations to Solve the Distal Reward Problem

When a reward occurs, several previous cues and actions are, in general, equally likely to be the cause. One trial is therefore not enough to understand the correct relationship. When more trials are attempted with variable conditions, the responsible cues and actions will be invariant and always present, whereas the disturbing and unrelated cues and actions may change from trial to trial. How can a neural network discern, over multiple trials, which stimuli and actions lead to rewards, and which are instead unrelated? Secondly, how can the network make the association despite the temporal gap, or delay, between stimuli, actions, and rewards?

Eligibility traces (Wang et al., 2000; Sarkisov and Wang, 2008) or synaptic tags (Frey and Morris, 1997; Redondo and Morris, 2011) are synapse-specific values with relatively slow dynamics believed to express the *eligibility* of a specific synapse for later changes. The duration of traces must be at least as long as the delays between cues, actions, and rewards. A reward is generally conveyed by means of a modulatory signal (Montague et al., 1996; Farries and Fairhall, 2007; Florian, 2007; Porr and Wörgötter, 2007; Soltoggio et al., 2008; Pfeiffer et al., 2010). However, when rewards are delayed, the neural activity that caused such reward is not present anymore. When rewards are delayed, modulation cannot act on the current neural activity, because that may not be related to the present reward. In such cases, it makes sense that modulation multiplies the eligibility traces to give a weight update. Such a modulatory signal changes the synaptic weights of those synapses that are eligible, and leaves the other synapses unchanged (Izhikevich, 2007; Päpper et al., 2011; Soltoggio and Steil, 2013). One fundamental and open question in this approach is what rule promotes or downgrades synapses to be eligible or ineligible at any time. Izhikevich (2007) uses the precise spike-timing to create traces according to a traditional STDP rule. Alternatively, the principle of rare correlations (Soltoggio and Steil, 2013), also used in the present study, prescribes that spiking neurons are not necessary so long as traces express correlating events and are created parsimoniously. The fundamental aspects in the creation of traces is the maintenance of a low balance of traces with respect to the overall number of synapses. Those rare traces allow the network to isolate the reward-triggering synapses in a few trials. The decay time of traces is related to their production rate, in a way that longer-lasting traces can be maintained if the rate of production is further decreased. By means of this balance, rewards with longer delays can be correctly associated with previous cues and actions.

The principle is illustrated by the following example. Assume that in a relatively small network with 100,000 synapses, high activity across one single synapse σ triggers a reward. Such a reward, however, is delivered with a delay between 1 and 3 s. Assume that correlations between connected neurons across the whole network are 1%/s of the total number of synapses. Those correlations generate eligibility traces at the specific synapses. If the traces have a time constant of 1 s, they decay exponentially and are negligible after 3 s. Therefore, at any time, approximately 3,000 synapses are eligible (i.e., 3% of the total). When correlating activity across σ triggers a reward, which is conveyed as a modulatory signal to the whole network, the reward episode reinforces approximately 3,000 synapses (the eligible synapses). In other words, the synapse σ caused a reward, but because the network is not silent and because the reward is delayed, thousand of other synapses also carried correlating activity before the reward delivery. If σ carries correlating activity more times, and more rewards are delivered, each time approximately 3,000 random synapses are reinforced. Only σ, because is the reward-triggering synapse, is reinforced consistently. Other synapses that are reinforced consecutively by chance become fewer and fewer at each reward episode. The number of synapses that are reinforced twice consecutively is the 3% of 3%, i.e., 0.09%, or 90 synapses from a total of 100,000. After only four reward episodes, 0.03^4^ = 0.0027%, i.e., three or fewer synapses have been reinforced consecutively. By the fifth reward episode, σ is likely to be the only synapse that was reinforced consistently. Thus, the use of rare correlations allows for a logarithmic-like search among noisy and spontaneous network activity where one single synapse among hundred of thousand triggers a reward. For more detail of this experiment, see (Soltoggio and Steil, 2013).

If correlations are not rare, e.g., 10%/s of the total or more, too many synapses are reinforced at each reward episode, causing some synapses to reach high values even when they are not triggering a reward. The rarer the correlations, the fewer are the unrelated synapses that are reinforced, and therefore the learning is more precisely targeted to the reward-triggering synapses. On the other hand, extremely rare correlations results in a network that selects synapses for reinforcement on a very sporadic basis, thereby resulting in a robust but slower learning.

The principle of rare correlations leads to the question of what rule can be used to extract them from the neural activity. The rarely correlating Hebbian plasticity (RCHP) was proposed in Soltoggio and Steil (2013) to address this question. This mechanism, described in detail in the next section, is employed for the first time in this study with a neuro-robotic experiment to learn associations of stimuli, actions, and rewards.

### 2.1 Rarely correlating hebbian plasticity

The Rarely Correlating Hebbian Plasticity (RCHP) (Soltoggio and Steil, 2013) is a type of Hebbian plasticity that filters out the majority of correlations and produces non-zero values only for a small percentage of synapses. Rate-based neurons can use a Hebbian rule augmented with two thresholds to extract low percentages of correlations and decorrelations. The RCHP rule is expressed by

(1) $$\text{RCH}{\text{P}}_{ji}\left(t\right)=\left\{\begin{array}{cc} +\alpha \; & \text{if}\;v_{j}\left(t-t_{pt}\right)\cdot v_{i}\left(t\right)>\theta_{hi}\\ +\beta \; & \text{if}\;v_{j}\left(t-t_{pt}\right)\cdot v_{i}\left(t\right)<\theta_{lo}\\ 0\; & \text{otherwise}\\ \; \end{array}\right.$$

where *j* and *i* are a presynaptic and a postsynaptic neuron, α and β two positive learning rates (in this study set to 0.1) for correlating and decorrelating synapses respectively, *v*(*t*) is the neural output, *t_pt_* is the propagation time of the signal from the presynaptic to the postsynaptic neuron, and θ*_hi_* and θ*_lo_* are the thresholds that detect highly correlating and highly decorrelating activities.

The rule expressed by equation (1) has two main features. The first is that the majority of neural activity does not correlate. Only a small percentage of synapses, determined by the thresholds θ*_hi_* and θ*_lo_*, has correlating values different from zero. This feature makes the RCHP different from a classical Hebbian rule in which all activity correlates along a continuous spectrum of values. A neural model that modulates classical Hebbian plasticity changes all synapses to a various extent because all synapses that carry non-zero activity are expected to correlate. Such an overall weight change can potentially wipe existing neural connections without reinforcing sufficiently those synapses that are responsible for a reward. On the contrary, the RCHP rule extracts a small percentage of synapses to be eligible for a weight update, leaving the majority of synapses unchanged and stable. A second feature of the RCHP rule is that detected correlations attempt to capture the cause-effect relationship of signal propagation across synapses. Similarly to STDP, when a high presynaptic activity value leads to a high postsynaptic activity value, the event is captured by the RCHP rule. In fact, the activity of the presynaptic neuron at time *t* is multiplied by the activity of the postsynaptic neuron at time *t* + *t_pt_*, which is the time when the signal from the presynaptic neuron reaches the postsynaptic neuron. It is later explained that the propagation time and sampling time can be equivalent. In this way, the time window for detecting a correlation is effectively one time step.

The thresholds θ*_hi_* and θ*_lo_* are estimated online to target an average rate μ of approximately 0.5%/s of rare correlations. θ*_hi_* and θ*_lo_* are assigned initially arbitrary values of 0.1 and −0.1 respectively. A first-in first-out queue of correlations *cq*(*t*) holds the number of correlations registered at each step during the recent past (in this implementation for the last 10 s). If the number of measured correlations during the last 10 s is higher than 5 times the target μ, i.e., higher than 2.5%, θ*_hi_* is increased of a small step η = 0.002/s. If the correlations are too few, i.e., less than 1/5 μ (0.1%), the threshold is decreased of the same small step. The same procedure is applied to estimate θ*_lo_*. It is important to note that such a procedure is an heuristic devised to implement a rudimentary homeostatic mechanism to extract rare correlations. The precise parameters used to implement the homeostasis are not particularly crucial as long as correlations are rare on average. In fact, the instantaneous rate of correlations and the long term dynamics vary considerably according to fluctuations of the neural activity, various input regimes, and weight changes. The self-tuning of the thresholds, as it is used in the present algorithm, is not meant to be a precise rule, but it is devised to ensure that, on average, only rare correlations are detected throughout the neural network. The large majority of synapses carry activity across neurons that do not correlate. A summary of the algorithm above is provided in the Appendix 6.

### 2.2 A neural model with eligibility traces and modulation

The RCHP rule acts on eligibility traces *c_ji_* on each synapse between a presynaptic neuron *j* and a postsynaptic neuron *i*. A modulatory signal *m*, which is governed by a fast decay and by the exogenous input reward *r*(*t*), converts eligibility traces to weight changes. The changes of the eligibility traces *c_ij_*, weights *w_ij_*, and modulation *m* are governed by

(2) $$\begin{array}{rcl} {ċ}_{ji} & =-c_{ji}∕\tau_{c}+\text{RCH}{\text{P}}_{ji}\left(t\right) & \end{array}$$

(3) $$\begin{array}{rcl} {ẇ}_{ji}\left(t\right) & =m\left(t\right)\cdot c_{ji}\left(t\right) & \end{array}$$

(4) $$\begin{array}{rcl} ṁ\left(t\right) & =-m\left(t\right)∕\tau_{m}+\lambda \cdot r\left(t\right)+b. & \end{array}$$

where a reward episode at time *t* sets *r*(*t*) = 1, which increases the value of *m*(*t*) proportionally to a constant λ. A baseline modulation *b* can be set to a small value and has the function of maintaining a small level of plasticity. The modulatory signal decays relatively quickly with a time constant *τ_m_* = 1 s, while traces have τ_*c*_= 4 s. The neural state *u_i_* and output *v_i_* of a neuron *i* are computed with a rate-based model expressed by

(5) $$\begin{array}{rcl} u_{i}\left(t\right) & =\underset{j}{\sum }\left(w_{ji}\cdot v_{j}\left(t\right)\cdot \kappa_{j}\right) & \end{array}$$

(6) $$\begin{array}{rcl} v_{i}\left(t+\Delta t\right) & =\left\{\begin{array}{cc} \text{tanh}\left(\gamma \cdot u_{i}\left(t\right)\right)+\xi_{i}\left(t\right)\; & \text{if}\;u_{i}\geq 0\\ \xi_{i}\left(t\right)\; & \text{if}\;u_{i}<0\\ \; \end{array}\right. & \end{array}$$

where *w_ji_* is the connection weight from a presynaptic neuron *j* to a postsynaptic neuron *i*; κ*_j_* is +1 and −5 for excitatory and inhibitory neurons respectively to reflect the stronger effect of less numerous inhibitory neurons; γ is a gain parameter; ξ*_i_*(*t*) is a uniform noise source drawn in the interval [−0.1,0.1]. The sampling time is set to 200 ms, which is also assumed to be the propagation time *t_pt_* [equation (1)] of signals among neurons. The values of all parameters are specified in Appendix 6. The architecture of the network with the inputs and outputs is outlined in the next section.

## 3 Conditioning in a Human-Robot Interaction

The principle of rare correlations is applied to a network model to perform classical and operant conditioning with the robotic platform iCub. The robot iCub and the hardware set-up are described in the following section. The classical and operant conditioning scenarios are illustrated in Sections 3.2 and 3.3. The learning networks with the inputs and outputs are described in Section 3.4.

### 3.1 The robotic platform

**Figure 1:**
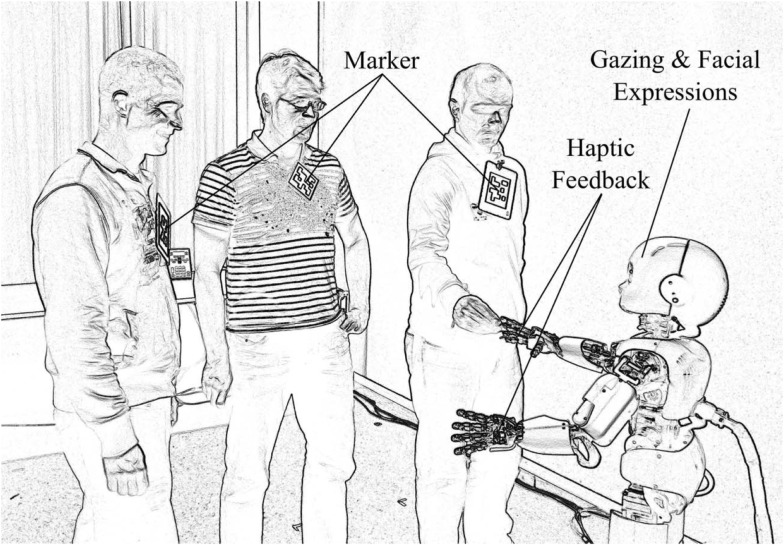
**The humanoid robot iCub in the experimental environment**. The robot detects people in its field of view with the help of markers. Haptic sensing delivers rewarding or punishing signals to the learning networks. Gazing by means of head movements, speech output, and facial expressions provide feedback to the human participants.

The iCub is a child-sized humanoid robot of 90 cm of height, weighing 23 kg, and comprising 53° of freedom (Tsakarakis et al., 2007). Figure 1 shows a rendered photo of the iCub interacting with people in the experimental environment. The robot facilitates human-robot interaction by means of haptic sensors in the hands, cameras, and its capability to display facial expressions. Expressions are produced by means of light-emitting diode arrays below the shell of the head. The position of the eye lids also add expressivity. In the current study, the facial expressions are limited to neutral, happy, and sad. Synthesized speech is produced via speakers mounted at the robot rack and it is used in the current scenario to provide additional feedback.

Cameras in the artificial eyes provide visual information of the surroundings. The visual input is used to detect people and objects in the room. In particular, markers are attached to people to make them easily identifiable (Figure 1). Additionally, object trackers signal the appearance of colored balls in the visual field of iCub. Additional details on the type and meaning of the inputs and outputs are explained in the following sections.

### 3.2 Learning who is the tutor (classical conditioning)

This experimental scenario aims at testing the capability of the proposed network model to perform classical conditioning in a realistic human-robot interaction.

The robot monitors the environment moving his head and shifting its gaze over the room. This movement has the purpose of enlarging the field of view and endowing the iCub with a naturally looking behavior. The iCub is capable of recognizing different people identified by markers. Of all the people taking part in the experiment, one particular person is designated to be the tutor. The tutor is a person who takes care of the iCub, and signals that by conveying an haptic input with the touch of the iCub’s hand. This signal represents an unconditioned stimulus that triggers an *innate*, i.e., pre-wired and fixed, positive reaction. Such a reaction corresponds also to a burst of modulatory activity as described in following sections. The haptic input can be interpreted as the delivery of food to Pavlov’s dog. The iCub reacts to the unconditioned stimulus displaying a smiling face expression and saying positive sentences like “Thanks,” or “I like it.” The expression of a positive state, which follows an unconditioned stimulus, is always related to a burst of modulatory activity. While the iCub is constantly aware of a number of people in the room (as shown in Figure 1), from time to time the tutor enters the room and touches the hand of the iCub, thereby causing a positive smiling reaction.

In classical conditioning, if a stimulus predicts consistently the delivery of a reward, the learning process leads the agent (in this case the robot) to react immediately when the tutor enters the room, before any actual reward is given. The experiment in this scenario tests the learning capability of the proposed network model to associate a conditioned stimulus (CS) to a reward, also in the presence of a number of other disturbing stimuli.

### 3.3 Learning the colors (operant conditioning)

A second scenario aims at testing operant conditioning, an experiment in which the iCub learns by trial and error to pronounce the correct word corresponding to the color of objects. The operant conditioning phase follows the classical conditioning only for practical reasons. When the iCub has learnt to recognize a tutor, it can easily follow his/her position and track colored objects. When the iCub detects a color object, it pronounces the name of a color. Initially, such an action is random because the iCub has no knowledge of which color corresponds to which name. If the color is correct, the tutor awards the iCub with a touch to the right hand, which delivers a reward to the network. If the iCub guesses the wrong color, the tutor ignores the answer and tries again after a few seconds. The cue (i.e., the colored object) and the action (i.e., the enunciation of a color) are not present anymore when the tutor gives the feedback. Thus, the neural mechanism that associates past actions with present rewards is tested in this scenario.

**Figure 2:**
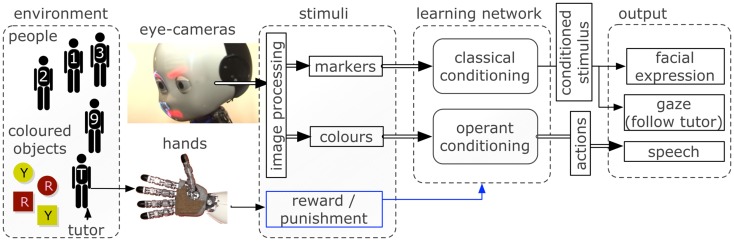
**The robotic software and hardware architectures used to interface the environment with the learning neural network**. Inputs are captured via cameras and haptic sensors and processed to provide a vectorized representation to the network. The control network triggers facial expressions, gazing behavior, and speech output depending on the neural activity.

A scheme of the inputs and outputs in the robotic scenario is shown in Figure 2. The details of the learning network are explained in the next section.

### 3.4 The learning networks

The central controller comprises two neural networks, one for classical, and one for operant conditioning. The networks do not differ qualitatively because the modulated RCHP is capable of both operant and classical conditioning. However, due to the diverse type of inputs and outputs in the two tasks, the two networks represent effectively two separate areas of a neural system.

Each network has 800 excitatory neurons and 200 inhibitory neurons whose activity and outputs are governed by equations (2) and (3). Each neuron is connected to another neuron with probability 0.1. All excitatory neurons have plastic afferent connections that vary in the interval [0, 1] according to equation (3). Inhibitory neurons have fixed afferent connections. The network has therefore a random connectivity and random initial weights.

**Figure 3:**
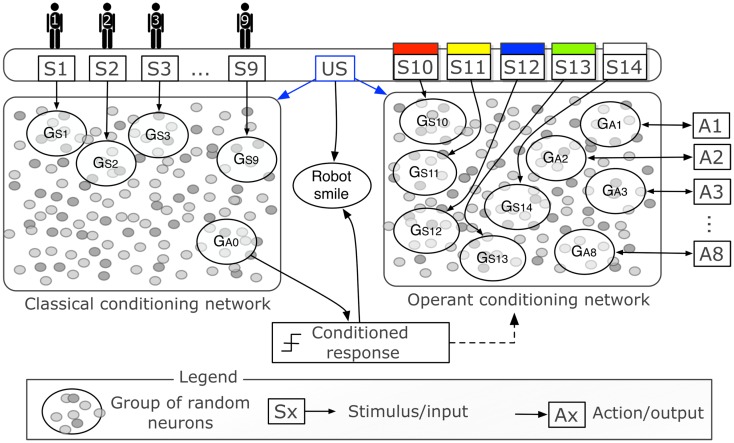
**Graphical representation of the control networks (expansion of central part of Figure 2)**. Two networks of 1,000 neurons each represent two distinct neural areas to perform classical and operant conditioning. The two networks differ only in the inputs and outputs, and in the initial random connectivity. The binary stimuli *S*1*‥S*9 indicate the presence of different people in the visual field of the iCub and are delivered to their respective groups of random neurons *G*_*S*1_‥*G*_*S*9_. The binary stimuli *S*10‥*S*14 indicate the presence of objects of five different colors (all five colors were tested in simulation, only two, S10 and S11, with the real robot). The actions *A*1‥*A*8 correspond to the enunciation of one particular color. The haptic sensor delivers a reward that represents the unconditioned stimulus (US). Both the US and high activity of *G*_*A*0_ cause the robot to smile.

Figure 3 is a graphical representation of the two networks with the inputs and outputs. Each person-stimulus (*S*1*‥S*9) is conveyed to the network by increasing the neuron state *u* by 10 for each neuron in a group of 60 randomly selected excitatory neurons (*G*_*S*1_‥*G*_*S*9_). The activity of one group of neurons (*G*_*A*0_), composed of 60 randomly selected excitatory neurons, triggers the conditioned response, i.e., it becomes active when the tutor is recognized after conditioning. The activity of a group is computed as the sum of the output of all neurons in the group, normalized by their number. Both networks receive a modulatory signal when the unconditioned stimulus is given by touching the iCub’s hand. The haptic sensor conveys a modulatory signal that acts in the network as the signal *m* in equation (3).

Neurons in input groups do not receive connections from the rest of the network. Such a topology is devised in the current study to cope with real-world persistent and simultaneous input signals. In fact, as opposed to Izhikevich (2007) and Soltoggio and Steil (2013), in which stimuli were brief and impulse-like in nature, the network in the current experiments may receive continuous stimuli for long periods and simultaneously. Such input regimes, combined with Hebbian-driven growth of recurrent loops, might induce self-sustained activity, an unwanted regime in which neural dynamics do not respond to input anymore. This topology assumption prevents such a problem and is compatible with the role of input neurons.

The color trackers send inputs to the operant conditioning network. These binary signals are injected raw and unprocessed in the network through the groups of neurons *G*_*S*10‥*S*14_. As opposed to the classical conditioning network, which has only one output, the operant conditioning network has eight different outputs, corresponding to eight possible actions, i.e., the enunciation of the name of eight different colors. Neurons in the output groups do not project recurrent connections to the network. Such a topology is important to prevent that high neural activity generated by actions is feed unnecessarily back to the network. When a color-stimulus is present, the activity levels of the output groups are monitored for 1 s. If none of the groups reaches 30% of the maximum activity at the end of the waiting period of 1 s, many groups might have nearly equivalent levels of activity. In other words, when weights are low, the network may not be able to express a clear decision on what action to perform. To overcome this situation, the group with the highest activity, even by a small margin, triggers the action, which in turn increases the activity of its group and lower those of the other groups (*u* is increased/decreased by 10). This change in the neural activity is in effect an action-to-network feedback meant to inform the network of which action was performed. These dynamics are similar to winner-take-all policies (Kaski and Kohonen, 1994). In this way, the network can correlate correctly the input group with the action group that corresponds to the action performed.

The two networks are independent and can be tested independently. Nevertheless, the conditioned stimulus in classical conditioning, i.e., the tutor, is used to start the second learning phase that tests operant conditioning. When the group *G*_*A*0_ responds with high activity, signaling the presence of the tutor, the robot switches to operant conditioning with a probability 0.1/s. This behavioral sequence is not a central feature of the experiments but creates a natural interactive sequence of actions, which allows the participants and the tutor to observe both classical and operant conditioning taking place.

## 4 Experimental Results

The experiments in this section test the learning capabilities of the control network both with the iCub robot and in simulation. The control network is simulated with the Matlab scripts provided as support material. The experiments were also video recorded. Both Matlab scripts and the illustrative video can be downloaded at the author’s associate website http://andrea.soltoggio.net/icub. The robotic experiments require a real robot, or a robot simulator. The Matlab code can be also used as a stand-alone script with simulated input/output flow. The simulation without a real robot is used to test precisely controlled input-output regimes and timing which are difficult to achieve in a real-life human-robot interaction.

### 4.1 Classical conditioning

The experiments in this section test the classical conditioning scenario previously described in Section 3.2. The experiments are conducted with the iCub. Further tests in simulation are also presented.

#### 4.1.1 Real robot conditioning

The experiment was conducted by instructing nine people to approach the iCub and remain in its visual field for a random amount of time between a few seconds and approximately 1 min^1^. The participants did not follow a particular pattern in coming and leaving, and simply approached the robot, like visitors could do in an open exhibition, fair, or museum. Each person was uniquely identified by a marker as in Figure 1 and corresponded to one stimulus in the range *S*1*‥S*9. The participants could freely move in front of the robot and were not instructed to perform particular actions. The tutor also entered and left the robot’s field of view at random times. As opposed to other people, the tutor also touched the iCub’s hand each time he approached the robot, thereby delivering a reward. Such rewards were delivered at random times by the tutor without a precise pattern. Other people, beside the tutor, could be present at the time of reward, making it difficult to establish the correct association between the tutor and the reward.

**Figure 4:**
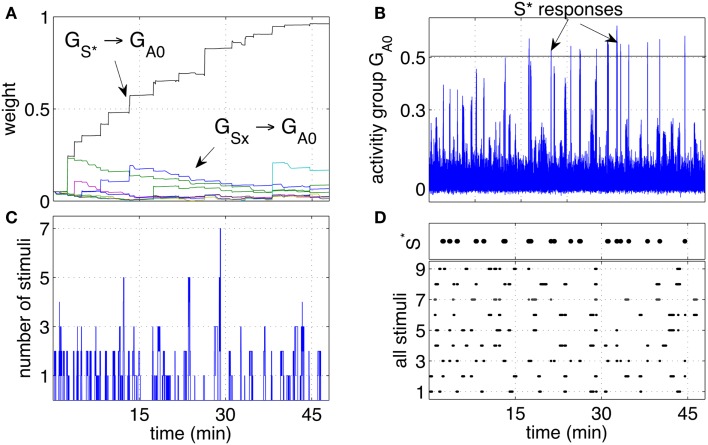
**Classical conditioning with disturbing stimuli**. **(A)** The average strength of all weights connecting neurons in the groups *S*1*‥S*9 to neurons in the output group *G*_*A*0_ are shown. The pathway S* → *G*_*A*0_ grows to reach the saturation value. The other pathways remain at low values with only occasional increments. **(B)** The activity of the output group *G*_*A*0_ is characterized by increasingly high peaks. Those peaks are the learnt responses to the stimulus S*. **(C)** Number of simultaneous stimuli present at any time. The plot shows that at times the network receives many stimuli simultaneously, making it difficult to detect which stimulus is causing a reward. **(D)** The presence of all the stimuli *S*1*‥S*9 and S* is plotted to show the correspondence of the arrival of S* with the peaks of the neural activity **(B)**.

Over time, the pathway that connected $G_{s*}$ (the neuron group that receives stimuli when the tutor is present) to the group *G*_*A*0_ grew consistently stronger. The pathways connecting the other groups *G_Sx_* grew only marginally and not consistently as shown in Figure 4A. The growth of the pathway $G_{sx}$ to *G*_*A*0_ led to an increased response of the group *G*_*A*0_ to the stimulus S* as shown Figure 4B. While the stimulus S* initially did not elicit a particular response, with time and more rewarding episodes, the network started responding with significant peaks in the activity when the stimulus S* was perceived. Between the 7th and the 9th reward episode, and approximately after 20 min, the activity of *G*_*A*0_ presents distinct peaks in response to S*. When the activity of the output group reached a preset threshold of 0.5, it caused a conditioned response. The response consisted in a smiling expression and a phrase like “Hello, it’s nice to see you again,” or “Hello, you are my friend.” These sentences were so structured to manifest the conditioned response, representing effectively a reward prediction. As with the unconditioned response, the iCub smiled. The robot was also pre-programmed to follow the tutor’s position with head movements to express clearly that the recognition had occurred.

Repeated experiments showed that the learning is manifested in three phases. An initial phase in which the tutor is not being recognized, an intermediate phase in which the tutor is recognized at times, or with a delay, and a final phase in which the tutor is recognized consistently and without delay. The intermediate phase is caused by the noisy fluctuations in the neural activity. When the pathway from $G_{s*}$ to *G*_*A*0_ is not yet strong, such fluctuations result in inconsistent or delayed responses.

The activity of *G*_*A*0_, after learning takes place, becomes a predictor of a reward delivery. The conditioning occurs despite two potential obstacles that derive from the real-life robotic scenario, and namely, (1) the noisy and unreliable perception of cues, and (2) the presence of many cues at the same time. In particular, the detection of markers is not 100% reliable for a number of reasons. Affecting the reliability of the detection are varying light conditions, different orientation of the markers due to the free movement and orientation of the participants, the obstruction of markers and noise in the camera. The slow decay of eligibility traces however ensures that the presence of a stimulus, in the present or in the immediate past, is represented at the synaptic level by the traces themselves. As a result, imprecise, unreliable, and noisy perception does not compromise the neural learning dynamics. The simultaneous presence of the reward-predicting stimulus and other disturbing stimuli is a potential obstacle in learning. Figure 4C shows that many stimuli are often present simultaneously. This situation induces occasional reinforcement of disturbing stimuli, as can be observed in Figure 4A. Nevertheless, the network reinforces consistently only the reward-predicting stimulus. Figure 4D shows the time of arrival of all nine stimuli and the correspondence of S* with the intense network responses in Figure 4C. The experimental results in this section show that the control network, embedded within the robotic platform and exposed to human-robot interaction, modifies the connection weights to implement classical conditioning.

#### 4.1.2 Simulated input/output flow

The previous experiment can be run as a stand-alone script in Matlab without the interface with the robot. In the simulated version, the signals representing the people are generated by means of a Poisson process that ensures random patterns in the sequence of stimuli. Thus, the experiments in this section eliminate possible bias in the pattern of appearance of people and tests rigorously the neural learning. The stand-alone experiments offer the possibility of reproducing the results with the provided Matlab scripts without a robot.

**Figure 5:**
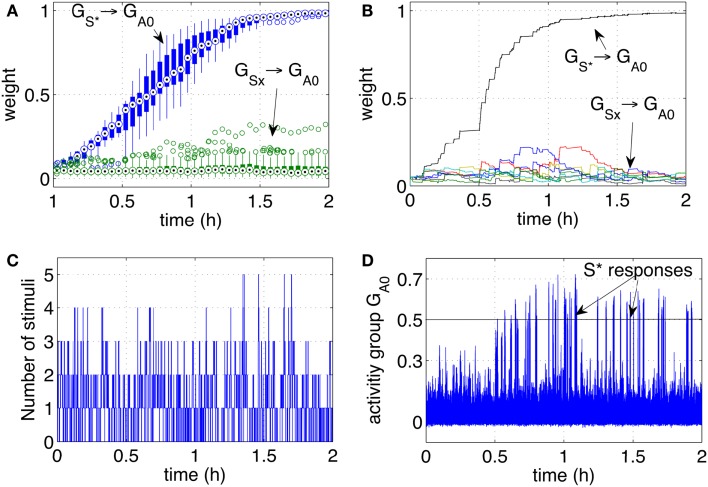
**Classical conditioning with disturbing stimuli and simulated input sequence**. **(A)** The connection strengths of the pathways from the input groups *G*_*S*1‥*S*9_ to the output group *G*_*A*0_ are shown here. The statistics include a set of 10 experiments and are represented by box plots indicating the median (central point), 25th and 75th percentiles (thick lines), most extreme data points (thin lines), and outliers (circles) (McGill et al., 1978). The box plots are computed and drawn over 3-min intervals. The strength of the pathway $G_{s*}\to G_{A0}$ (10 lines from 10 runs) grows consistently during the learning process and across all independent runs. The pathways from disturbing stimuli (box plots from eight lines for each run, i.e., 80 lines) remain at low values. **(B)** The strength of the pathways as in **(A)** are shown in one particular run. **(C)** Number of stimuli present at any time during one particular run. **(D)** The activity of the output group *G*_*A*0_ during one particular run. The network increases its responses as the stimulus S* becomes progressively associated with the reward.

Each stimulus (representing one person) has a probability 0.15%/s of appearing, i.e., all stimuli are independent and may be present at any time. Once present, one stimulus lasts for a variable interval in the range [3, 30] s. As before, one particular stimulus S* ∈ (*S*1*‥S*9) is designated to be the rewarding stimulus. When S* is present, it causes a reward to be delivered in a random interval [0, 5] s. The simulation was run extensively for 2 h to test the stability of the learning, and to observe in particular that the pathways from the disturbing stimuli remained low. To assess further the robustness of learning, 10 independent runs were executed. Figure 5A, shows the statistical analysis of the pathways of all 10 independent runs. Figures 5B–D show respectively the weight changes, the number of stimuli and the network activity for one particular run. The results are qualitatively similar to the robotic experiment that was conducted with human subjects interacting with the robot. This indicates that differences in timing of the reward, duration, and frequency of stimuli between robot and simulation are not affecting the learning dynamics. It can be concluded that, as hypothesized, uncertain timing of the stimuli and variable delays are successfully processed by the neural network to discover the correct cue-reward sequence.

#### 4.1.3 Delayed rewards after stimuli occurrence

In the previous experiments, the delivery of the reward occurs with a variable delay up to 5 s, but the causing stimulus S* is likely to be present at the moment of reward delivery, except for the flickering and view obstruction of the marker. This fact derives from the intrinsic nature of the scenario in which a person is visible to the robot while pressing its hand (Figure 1). However, the capability of solving the distal reward problem is demonstrated when the reward occurs with a delay after the stimulus has ceased. This is the scenario in which, for example, a brief noise or sound predicts the delivery of the reward seconds later (e.g., the bell in Pavlov’s experiment). To simulate this condition, in a variation of the original experiment, each stimulus remains present only for 1–2 s. The network receives a reward with a delay up to 5 s after the responsible stimulus has ceased. This experiment was run only in simulation. The equivalent version with the robot involves, for example, the recognition of a distinctive noise that predicts the arrival of each different participant.

**Figure 6:**
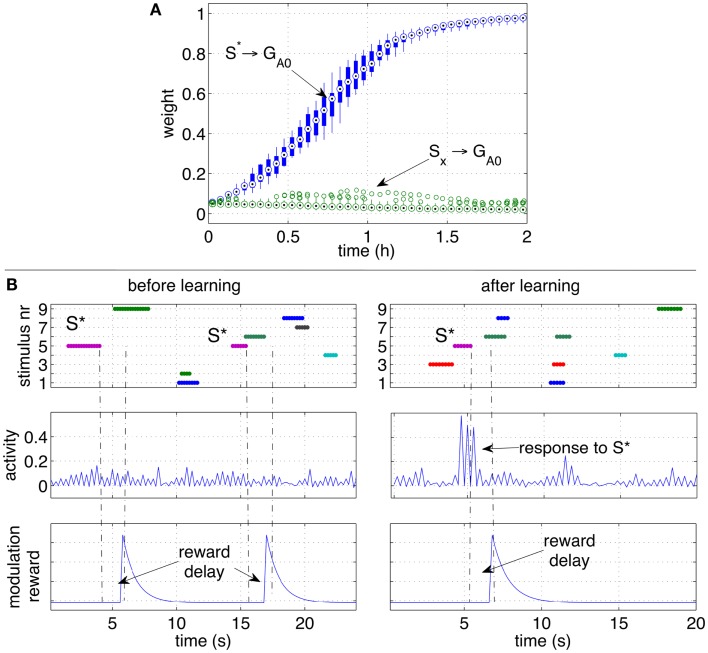
**Classical conditioning with delayed rewards after stimuli occurrence**. **(A)** The strengths of the pathways are shown with box plots over a set of 10 independent runs. Similarly to Figure 5A, the pathway from the conditioned stimulus to the output group increases consistently, while the other pathways from the other stimuli remain at low values. **(B)** A close-up over a brief simulation interval during a particular run. The stimuli (top row), activity of group *G*_*A*0_ (middle row) and the modulatory signal (bottom row) are plotted before (left) and after (right) learning. While S* does not elicit a response before learning, after learning S* causes a clear increase of the activity of the output group before the reward is delivered.

Also in this scenario, the network learns to respond to the CS S* despite S* is not present anymore at the moment of reward delivery, and other disturbing stimuli may be present instead. Similarly to the previous experiment, throughout the simulation the response of the output group *G*_*A*0_ grows stronger. Figure 6A shows that the strength of the pathways from S* to *G*_*A*0_ grows consistently in all the 10 independent simulations.

To observe the network behavior during a specific occurrence of the conditioned stimulus, Figure 6B shows the response of the output group to stimulus S* before and after learning. The graphs show that a reward is delivered when the stimulus S* is no longer present, and that disturbing stimuli may occur in between S* and the reward delivery. While S* initially does not elicit a response in the network, after learning, the neural activity of the neurons in the group *G*_*A*0_ is significantly higher than average. The peaks of activity in the right plot are a consequence of S* and occur before the reward is actually delivered (right plots). Note that the activity alternates between high and low values due to the effect of inhibitory neurons.

#### 4.1.4 The role of rare correlations and traces

The results in the previous sections showed robust learning dynamics in the classical conditioning scenario. How do rare correlations, eligibility traces, and delayed reward cooperate in the learning algorithm to achieve such a result?

**Figure 7:**
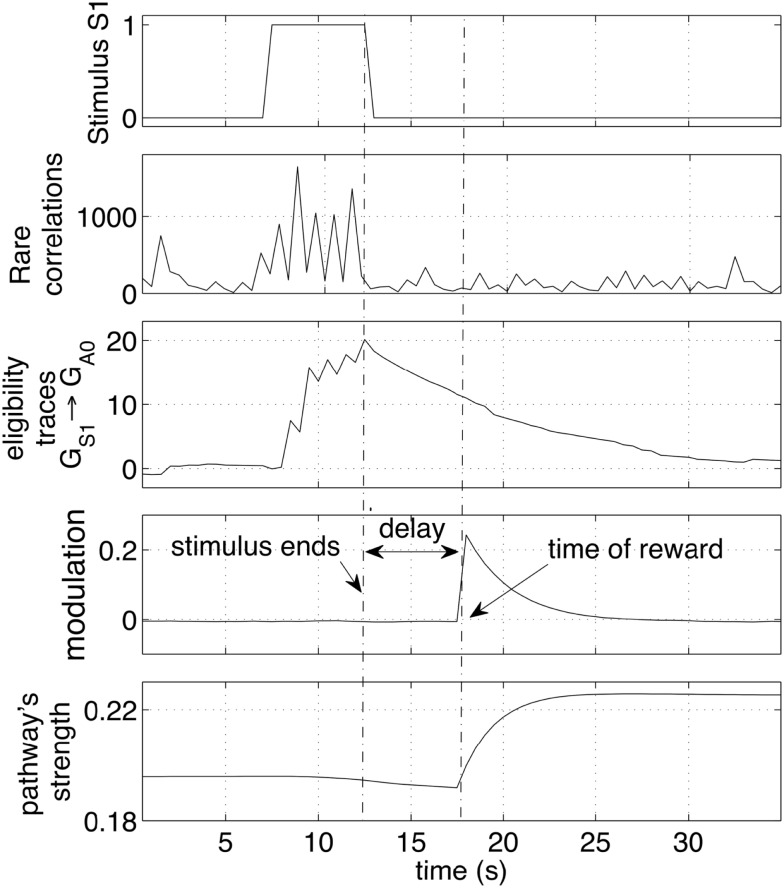
**Effect of a stimulus followed by a reward**. The sequence of stimulus, rare correlations, eligibility traces, reward and weight increase is shown during a 30 s interval. The network preserve a memory of a recent stimulus by selectively creating eligibility traces along the pathways that transmit signals. The traces are later converted to weight increase if a reward is delivered. The strength of the pathway is represented as the average weight.

This section looks at the small time-scale in which the weight changes occur. In particular, the neural dynamics are monitored and analyzed during a single cue-reward sequence. Figure 7 shows the arrival of a stimulus S1 (first row). Such an event is registered by the network with an increase of correlating activity (second row). Such correlations are concentrated mainly on connections from the group *G*_*S*1_ and generate a significant increase of the eligibility traces of those synapses (third row). Those eligibility traces then decay with a time constant of 4 s. When a reward is delivered a few seconds later, it multiplies the traces to produce a net weight increment. Note that the presence of traces causes a very small decrement of the pathway (bottom plot) before the reward is delivered. This decrement is due to the small negative baseline modulation given by the term *b* in equation (4). This setting causes a pathway to decrease its strength if repeated stimuli are never followed by a reward. It is important to note that all synapses in the network are active and transmit signals at all times. Nevertheless, because correlations are rare, other synapses in the network are affected by minor changes, resulting in negligible variations of the weights. The robustness to disturbances is ensured by the principle that on average only the reward-predicting stimulus consistently creates traces that are later converted to weight changes. Other stimuli cause also correlations and generate traces, but their values are not converted to weight changes.

### 4.2 Operant conditioning

Operant learning is triggered with probability 0.1/s when the iCub recognizes the tutor as a conditioned stimulus (CS) (after the robot was conditioned to recognize one person). At this point, the tutor presented different objects of different colors. Red and yellow colored objects were used with the robot. Up to five input colors were tested in simulation. Both real robot and simulation had eight actions available, i.e., eight output groups (*A*1*‥A*8) triggered the enunciation of eight colors.

Once the iCub detected a colored object, it enunciated the name of a color. If the color pronounced by the iCub correspond to that of the object, the tutor touched the right hand of the iCub, thereby providing positive feedback. If the iCub answered by enunciating another color, the tutor ignored the answer and waited for the next trial. Between each trial, the tutor waited a random amount of time, generally varying between 5 and 20 s. On an average trial, between a correct answer and the time the tutor touched the hand, a time between 1 and 3 s elapsed.

**Figure 8:**
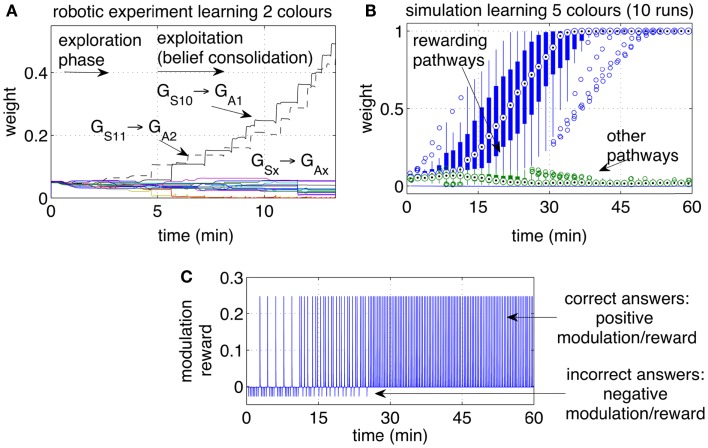
**Operant conditioning: learning stimulus-action associations**. **(A)** Example of one run with the robot learning two colors. The weights of the rewarding pathways (*G*_*S*10_ → *G*_*A*1_ and *G*_*S*11_ → *G*_*A*2_) and from the same inputs to all outputs are shown. After a period of exploration, the robot discovered the rewarding actions and repeated them consistently, causing the corresponding pathways to grow, while the other pathways remained at low values. **(B)** Weight dynamics during the simulated learning of five colors. The statistical analysis includes 10 independent runs. All correct associations (five colors for each of the 10 runs) were discovered within 30 min. All pathways that did not lead to a reward, i.e., the incorrect associations, remained at low values. **(C)** Modulation while learning five colors. Initially the modulation signal is negative due to negative rewards, a consequence of wrong answers. With time, each of the five color is associated with the correct response. When all answers are correct, all rewarding episodes become positive.

Initially the robot displayed an exploratory behavior. The exploration is due to neural noise and to the fact that none of the pathways is significantly stronger than the others. During the exploratory phase, the iCub answered with different colors each time the same object was presented, occasionally repeating the same color. The robot switched to choosing the correct answer after a few correct guesses. A higher level of reward, or a longer touch to the iCub’s hand, could be used to achieve a one-shot learning in which one single positive reward episode led to the repetition of that action, i.e., no further exploration. Figure 8A shows the strengths of the pathways from the two inputs *S*10 and *S*11 (representing two colors) to the actions (representing the enunciation of those colors). Each reward episode was caused by pressure on the iCub’s arm causing *r*(*t*) to be 1 during the touch.

In a variation of this experiment, the tutor could induce a small negative reward [*r*(*t*) = −0.5] by touching the left hand of the robot whenever a wrong answer was given. When that happened, the corresponding pathway registered a reduction in strength. At the next trial, the previous erroneous choice was therefore less likely to be selected, because the other pathways were stronger. These dynamics resulted in a faster exploration in which colors were not randomly selected: colors that resulted in negative reward were less likely to be named subsequently. The data from this experiment is not shown, but the simulated version described following adopts a similar rewarding policy.

The experiment with the iCub was extended in simulation to include five different colored objects (*S*10*‥S*14). The automated process produced one stimulus (corresponding to one colored object) every 20 s. Every stimulus was presented sequentially and circularly, i.e., in the sequence 1, 2, 3, 4, 5, 1, 2, …, etc. If the answer was correct, a reward *r*(*t*) = 5 was given with a delay in the interval [0, 5] s, otherwise a small negative reward [*r*(*t*) = −0.5)]was given. The weights of the pathways, statistically analyzed over 10 independent runs, are presented in Figure 8B. The plot indicates that within 30 min of simulated time, all objects during all runs were correctly associated with their respective colors.

It is important to note that the amount of weight increase depends on how much time elapses between the action and the reward. In the current study, exponentially decaying traces [equation (2)] were employed, making the trace decay over time as *e*^−*t*^. Because the modulation *m*(*t*) multiplies the traces to achieve a weight increment [equation (3)], the weight increase is also related to such a decay.

Interestingly, several tests showed that the answers became reliable when one pathway became approximately 20% stronger than the other pathways (measure only visually estimated). For smaller differences, stronger pathways were still more likely to drive the output, but the neural noise and random fluctuations in the neural activity meant that weaker pathways could at times prevail. When one pathway became at least 20% stronger than the others, the answer became reliable. Any further increase of such a pathway did not appear to manifest in a behavioral change. However, each increase in the rewarded pathways represents in effect a further consolidation of a behavior, which can be seen as a *belief* that stimulus S10, for example, is the color “red.” It can be inferred that in the phase of exploitation, the strength of the strongest pathway is an index of how *sure* or *confident* the robot is that the answer is the correct one. Although two or three correct and rewarded answers were sufficient to establish an immediate correct behavior, further trials provided confirmation, resulting in what can be named as *belief consolidation*. The effect of the weight strengths on behavioral properties such as exploration, exploitation, and belief consolidation is further investigated in the next section on behavior reversal.

As it is mentioned above, the operant conditioning phase was started conventionally by the recognition of the tutor. Nevertheless, the pathways in the network to the right of Figure 3, i.e., those that learn the colors, are learnt independently of the classical conditioning experiment. Once the colors are learnt, a new person may be introduced to the iCub as a new tutor. The iCub will be able to answer correctly to the new person because the recognition of the tutor is independent from the object-color associations.

### 4.3 Behavior reversal

In the previous section it was mentioned that the tutor could provide a negative reward touching the left hand of the robot. In effect, a negative reward (negative modulation signal) can be interpreted as a punishment. In this section, the use of punishment to implement behavioral reversal is tested.

In this new experiment, the tutor conditioned the iCub to learn one association between one color and the name of a color, as it was also done in the previous experiment. After the association was established, the tutor attempted to reverse this association by providing negative feedback. Each time the iCub was presented with the yellow object, and responded “yellow,” the tutor gave a punishment touching the left hand. A punishment was set to be equivalent to a reward but with opposite sign. The purpose of the tutor was to remove the previous association in favor of a new one. In this particular case, a whimsical tutor attempted to cancel the correct association “yellow” in favor of the enunciation “orange.”

**Figure 9:**
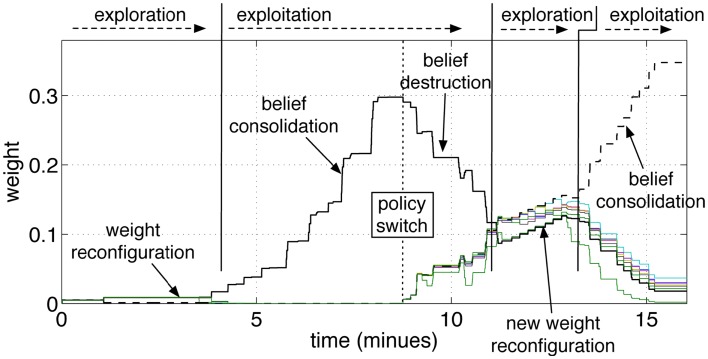
**Operant conditioning and behavior reversal**. The pathways from the input group *G*_*S*10_ to the action groups *G*_*A*1_‥*G*_*A*9_ are shown. As in the previous experiment, the robot attempts to guess the color during an initial exploratory phase. When the robot guesses the color correctly, and the tutor rewards it, the correct stimulus-to-action pathway is reinforced. Subsequently, the tutor changes his policy and gives a punishment if the robot perseverates with the previous answer. This leads to a convergence of all weights and to forgetting the previous association. A new exploratory phase then starts, which results in a new exploitation once the new correct color is guessed and the tutor rewards the robot.

Figure 9 shows the pathways from the group *G*_*S*10_ to the action groups. The graph shows the same initial phases of exploration and exploitation as in Figure 8. When the tutor starts giving negative feedback (marked in the graph with *policy* *switch*), the weights of the yellow-pathway decrease progressively. The reversal of the previously acquired behavior is gradual. The amount of negative modulation was in effect equal to the amount of positive modulation. Each punishment resulted in a decrement of the pathway comparable to the increment that was previously obtained by one rewarding episode. If the robot was previously rewarded many times and had established a strong association between a cue and one action, it was consequently more adamant to changes. As anticipated, it can be said that the strength of a pathway reflects a level of *belief*. A strong pathway, reflecting a strong belief, also resulted in a robust behavior in front of false or misleading, but occasional, input cues. Even if the robot received a punishment from a correct answer, for example due to an error or a whim of the tutor, the single episode did not reverse the robot belief unless the tutor insisted on the new policy.

The repeated punishments led the network to reduce progressively the difference in weights among the pathways. When all pathways reached similar values, the answers started to vary among colors, i.e., the robot resumed an exploratory phase. A new association was now possible. When the robot, seeing a yellow object, pronounced the correct color (orange, according to the new tutor’s policy), the tutor gave rewards and led the robot to build the new association, as reflected by the growth shown in Figure 9 at the end of the experiment. The length of time that is necessary to achieve the behavior reversal depends on the strength of the pathway (also indicating the strength of the belief) and the plasticity rate. Strong pathways and slow plasticity rates result in robust and slow-changing behaviors, while weak pathways and fast plasticity rate result in quick behavior reversal.

## 5 Discussion

The human-robot interactions presented in this study allowed human operators to explore the dynamics of learning in a natural scenario. The tests revealed a number of significant aspects of the neural model that can be compared to biological counterparts.

The generation of eligibility traces by means of rare correlations is a mechanism that selects synapses that may reflect relationships between stimuli or stimuli/actions. The event of a subsequent reward reinforces synapses that are even more likely related to a reward. The presence of disturbing stimuli and delays means that one reward episode is not sufficient to determine uniquely the stimulus that predicts a reward, or the action that causes it. Accordingly, the plasticity rule increases significantly the weights only over many consecutive rewards episodes, suggesting that a correct rate of learning is fundamental in conditioning experiments. A comparison of different learning rates was not rigorously conducted in the present study. Nevertheless, preliminary experiments confirmed the intuitive notion that fast plasticity rates result in a belief being established in fewer episodes. Fast plasticity rates, also possible in the proposed algorithm^2^, can be used to observe the accidental response-contingency hypothesis of Skinner (1948). Thus, superstitious behavior can be reproduced with the current model if weights are highly plastic, confirming that high learning rates may results sometimes in establishing wrong associations. However, while this position is a common assumption in machine learning, the proposed neural model attributes the causes of erroneous wrong associations to precise weight dynamics. The process of selecting synapses for weight update must be highly selective and the update must be moderate to endow the network with the necessary prudence before establishing an association. Further research in biology could ascertain whether, similarly to the present computational model, traces, and modulatory episodes in biological brains could be regulated parsimoniously to prevent runaway synapses (Hasselmo, 1994), forgetting (Wixted, 2004), or preserve learning capabilities (Anlezark et al., 1973; Hasselmo, 1999; Bailey et al., 2000; Reynolds and Wickens, 2002).

The decay rate of traces determines how long the network remembers a stimulus. Assume for example that the tutor shows the iCub a yellow object, to which the robot erroneously answers “blue.” The tutor ignores the incorrect response, but immediately, i.e., 1 or 2 s later, presents a red object to the robot that answers “red.” If now the tutor gives a reward, such a reward reinforces the association of the red stimulus to the red enunciation, but it reinforces to a small extent also the immediately preceding wrong association of the previous trial. If tutoring is enforced with insufficient time between trials, a correct learning is disturbed by interference with previous episodes. Interestingly, this interference is dealt with by the learning rule the same way as disturbing stimuli are, i.e., over the long term they are not reinforced as the reward-causing action. Such a consideration leads once more to the rate of learning: with slower learning rates, the learning is more robust to interferences. Unfortunately, even if in the long term slow learning rates guarantee better results, this behavior is generally not appreciated by the human tutor who might not display sufficient patience or perseverance toward a slow learning robot.

The test on behavior reversal showed that the weight dynamics in this experiment follow the *reconfigure-and-saturate* rule in Soltoggio and Stanley (2012), which describes the alternation of exploration and exploitation as a consequence of noisy anti-Hebbian plasticity (due to negative modulation and noise) and Hebbian growth (due to positive modulation). In that study, the strength of pathways also represented the probabilities of performing certain action. The growth and decrease of weights was not a consequence of weight tuning or memory decay, but, similarly to the present study, represented the consolidation or forgetting of behaviors. Whilst in Soltoggio and Stanley (2012) the reward was simultaneous with the actions, in the experiments of the current study the alternation of exploration and exploitation emerges from *delayed* negative and positive modulation. This confirms that the reconfigure-and-saturate dynamics in Soltoggio and Stanley (2012) can be reproduced also with delayed rewards as in the realistic robotic scenarios presented in this paper. In particular, the feedback-driven alternation of exploring and exploiting behaviors can be observed even with time gaps between causally related cues, actions, and rewards.

A behavior reversal can be induced, as in the presented case, by applying a negative reward, or punishment. However, the absence of a reward (or unconditioned stimulus) may also induce the extinction of actions (Gallistel, 1993). The absence of a reward is particularly relevant when there is an expectation after conditioning, e.g., food comes after pressing a lever. In the current experiments, expected reward is not modeled and the reward signal is used without pre-processing. A form of extinction is present in the current experiments because a small negative baseline modulation is present at all times [parameter *b* in equation (4)]. When a strong stimulus propagates through the network, it generates eligibility traces which make those pathways sensitive to modulatory signals for weight update. If no reward occurs in the following interval, the small baseline negative modulation causes also a small decrement of those synapses with high positive traces. Thus, extinction occurs if cues and actions are never followed by rewards. A fully fledged model of behavior extinction, including the modeling of an expected reward, was not the focus of the current study. A number of aspects must be clarified to introduce the notion of unexpected reward, or surprise. In particular, for each stimulus, an average value associated with previous rewards must be memorized in the network. Subsequently, a difference between expected and actual reward must be computed. However, if the timing of the reward is uncertain, it is also unclear when such a difference is to be computed. Moreover, the learning of a correct association may not require further reinforcement later on. In summary, the questions that emerge in scenarios with both delayed rewards and expected rewards make the topic a promising venue for extensions of the current model.

The current model does not implement blocking (Kamin, 1969). Blocking is a phenomenon in which, once a conditioned stimulus CS1 is associated with an unconditioned stimulus, a second conditioned stimulus CS2, occurring simultaneously to CS1, is not associated anymore. Simulations (not shown) indicated that a second stimulus (CS2) is also paired to the US. This characteristic, although different from some observations in animal learning (Kamin, 1969), shows the ability of the model of continuous learning and to discover new associations even after initial associations are established.

Finally, it is worth noting that the success in bridging temporal gaps emerges from the balanced equilibrium between the production rate of traces (by means of rare correlations) and their duration. In the current study, a time constant of 4 s for the eligibility traces was used. With such a constant, associations between cues and rewards can be discovered if a reward is delayed by a maximum of 10–12 s. Longer delays mean that the responsible stimuli and actions are forgotten. Making traces more durable, i.e., having a slower decay, is a way to empower a network to bridge even more distal rewards. To preserve the selectivity of the RCHP rule, longer-lasting traces must be compensated with a lower rate of production, i.e., they must be generated even more parsimoniously. Such a position suggests that long gaps between cues, actions, and rewards can be handled by a learning neural network only if the creation and destruction of traces is particularly rare (Soltoggio and Steil, 2013). For biological brains, which are notoriously subject to a considerably higher level of inputs and outputs, the current model predicts that particularly selective mechanisms could be responsible for filtering relevant information to be integrated later in time upon reward delivery.

## 6 Conclusion

This study demonstrates neural robotic conditioning in human-robot interactive scenarios with delayed rewards, disturbing stimuli, and uncertain timing. The neural dynamics employ rare neural correlations, eligibility traces, and delayed modulation to learn solutions in conditioning problems with realistic timing. The plasticity rule extracts rare correlations, generates eligibility traces, and uses them with Hebbian and anti-Hebbian plasticity according to environmental cues and human feedback. The result is robust classical and operant conditioning with delayed rewards and disturbances. The robotic experimentation proves the robustness and suitability of the proposed neural mechanism in learning with uncertain timing, unreliable inputs, delayed rewards, and variable human-robot reaction times and feedback.

This study also further promotes the idea that differences in the strength of neural pathways may reflect the tendency toward exploration or exploitation. Smaller differences cause the neural dynamics to be driven mainly by neural noise, which leads to exploration. Greater differences cause the network to exploit particular behaviors that were previously reinforced.

Finally, decaying eligibility traces model important learning dynamics with potential implications and predictions in biology. The model lends itself to predictions on how long and how many past events can be traced by a small network. Additionally, the plasticity rate and the strength of the pathways represent the rapidity with which a behavior (or a belief) is established, and the strength and robustness of such behaviors. Once a behavior is established, further confirmations and rewards continuously reinforce the involved pathways, thereby imprinting such a behavior that becomes later more difficult to eradicate. Such types of simulated behaviors are of interest in cognitive developmental robotics, an area in which delayed rewards and human interaction are used in learning processes. In conclusion, the proposed neuro-robotic model displays strongly bio-inspired synaptic and behavioral dynamics that are therefore relevant not only for robotics, but also for biology, neuroscience, and psychology.

## Conflict of Interest Statement

The authors declare that the research was conducted in the absence of any commercial or financial relationships that could be construed as a potential conflict of interest.

## Supplementary Material

The Supplementary Material for this article can be found online at http://www.frontiersin.org/Neurorobotics/10.3389/fnbot.2013.00006/abstract

## Footnotes

^1^In effect, it is not easy to impose an exact time to people entering and exiting the iCub’s field of view. The variability of such timing and overlapping of stimuli are characteristics of human-robot interactions.

^2^More plastic weights can be implemented in the current model with higher modulation, higher parameters α and β of the RCHP, or higher percentages of correlations. These factors are sometimes referred to in the literatures as “learning rate.”

## Appendix

### Details of the neural model

The plasticity rule (RCHP) described by equations (1, 2) and (4) is fully specified by the parameters in Table A1 in Appendix. The neural model described by equations (2–4) is fully specified by the values in Table A2 in Appendix. The integration of equations (2) and (4) with a sampling time Δ*t* of 200 ms is implemented step-wise by

(A1) $$\begin{array}{rcl} c_{ji}\left(t+\Delta t\right) & =c_{ji}\left(t\right)\cdot e^{\frac{-\Delta t}{\tau_{c}}}+\text{RCH}{\text{P}}_{ji}\left(t\right) & \end{array}$$

(A2) $$\begin{array}{rcl} m\left(t+\Delta t\right) & =m\left(t\right)\cdot e^{\frac{-\Delta t}{\tau_{m}}}+\lambda r\left(t\right)+b & \end{array}$$

**Table A1:** **Parameters of the plasticity rule (RCHP) and modulation**.

Time constant of eligibility traces [*τ_c_*, equation (2)]	4 s
α [Equation (1)]	0.1
β [Equation (1)]	0.1
λ [Equation (4)]	0.05 (0.07*)
*b* [Equation (4)]	−0.002/s
Target rate of rare correlations μ	0.5%

*(*) The higher value 0.07 is effectively a slight increase in the learning rate that was used in the classical conditioning experiment with brief stimuli (Section 4.1.3): this experiment set-up resulted in fewer rewarding episodes and so the higher value of λ led to convergence within the 2 h of simulated time*.

**Table A2:** **Parameters of the neural model**.

Excitatory neurons	800
Inhibitory neurons	200
Connection probability	0.1
Weight range	[0, 1]
Inhibitory weights	Fixed in [0, 1]
Excitatory weights	Plastic
Noise on neural transmission [ξ*_i_*(*t*), equation (6)]	Uniform [−0.1, 0.1]
Target rate of rare correlations μ	0.5%
Sampling time step [Δ*t*, equation (6)]	200 ms
Time constant of modulation [*τ_m_*, equation (4)]	1 s
Neural gain [γ, equation (6)]	0.25

The measured rates of correlations ρ*_c_*(*t*) and decorrelations ρ*_d_*(*t*) are computed over a sliding time window of 10 s summing all correlations and decorrelations buffered in *cq*(*t*) and *dq*(*t*)

(A3) $$\rho_{c}\left(t\right)=\Delta t\frac{\overset{t-10}{\underset{0}{\sum }}cq\left(t\right)}{10}\;,$$

and similarly for ρ*_d_*(*t*). The adaptive thresholds θ*_hi_* and θ*_lo_* in equation (1) are estimated as follows.

(A4) $$\begin{array}{rcl} \theta_{hi}\left(t+\Delta t\right)=\left\{\begin{array}{cc} \theta_{hi}+\eta \cdot \Delta t\; & \text{if}\rho_{c}\left(t\right)>5\mu \\ \theta_{hi}-\eta \cdot \Delta t\; & \text{if}\rho_{c}\left(t\right)<\mu ∕5\\ \theta_{hi}\left(t\right)\; & \text{otherwise}\\ \; \end{array}\right. & & \end{array}$$

and

(A5) $$\begin{array}{rc} \theta_{lo}\left(t+\Delta t\right)=\left\{\begin{array}{cc} \theta_{lo}-\eta \cdot \Delta t\; & \text{if}\rho_{d}\left(t\right)>5\mu \\ \theta_{lo}+\eta \cdot \Delta t\; & \text{if}\rho_{d}\left(t\right)<\mu ∕5\\ \theta_{lo}\left(t\right)\; & \text{otherwise}\\ \; \end{array}\right. & \end{array}$$

with η = 0.002. If correlations are lower than a fifth of the target or are greater than five times the target, the thresholds are adapted to the new increased or reduced activity. This heuristic has the purpose of maintaining the thresholds relatively constant and perform adaptation only when correlations are too high or too low for a long period of time.

The reward signal *r*(*t*) was impulse-like in nature for the simulated classical and operant conditioning experiments, i.e., lasting one computational step (200 ms). In the robotic experiments, the duration of the touch to the iCub’s hand/arm effectively determined the magnitude of the reward episode simply by making this signal last longer. The magnitude of *r*(*t*), in this study set in the range [1, 5], can be used to achieve different learning rates (data not shown).

The complete scripts for reproducing the experiment in simulation can be downloaded from the author’s associate website http://andrea.soltoggio.net/icub
